# The Hospital Nursing Supplement

**Published:** 1894-04-07

**Authors:** 


"The Hospital" Nursing Mirror.

Being the Extra Nursing Supplement of "The Hospital" Newspaper.

[Contributions for this Supplement should be addressed to the Editor, The Hospital, 428, Strand, London, W.C., and should have the word "Nursing" plainly written in left-hand top corner of the envelope.]

### News from the Nursing World.

### PRINCESS CHRISTIAN AT OXFORD.

Princess Christian visited the Sarah Acland Memorial Home for Nurses at Oxford last week, accompanied by Mrs. Max Müller, with whom she was staying. Her Royal Highness expressed her satisfaction with all she saw at the Home, and spoke to various patients and nurses.

### A NEW NURSES' LIBRARY.

The nurses of the London Hospital have long possessed a wonderfully complete and valuable collection of beautifully prepared "specimens" to aid them in their study of human physiology. It was presented to them by two of the consulting surgeons, Mr. Treves and Mr. Openshaw; and recently a fresh proof of the interest of the medical staff in the training school has been manifested—Mr. Rogers, with others of the residents, the committee and House Governor, have either by donations or by special gifts of books formed a nurses' library, with a nucleus of five hundred volumes, exclusive of the medical and nursing books, and every prospect of a great many more being shortly added. A first-class collection of medical and nursing, as well as general books of all kinds, is therefore provided for the present and future nurses and probationers of the London Hospital.

### TRAINED NURSES' ANNUITY FUND.

The Committee of this Fund, in consequence of a report of a sub-committee to whom the matter was referred, have decided not to hand over its funds to the Royal British Nurses' Association, but to continue the Fund on its own distinct lines and basis. The sub-committee, after conferring with Sir Henry Longley, the Chief Charity Commissioner, ascertained that the trustees of the Fund would not be justified in handing over the Trust Fund to another body on a resolution passed by a general meeting without the authority of the Court of Chancery or the Commissioners. The sub-committee failed to perceive any advantage to nurses in general which would be gained by the proposed amalgamation commensurate with the risk, if not the certainty, of diminishing the annual income, and thereby retarding the endowment of new annuities, as the sub-committee had reason to fear that the members would be by no means unanimous in favour of amalgamation, and that at least one, and he the largest contributor to the Fund, would in the event of its being carried withdraw from it altogether. Some years ago the Royal National Pension Fund demurred to a proposal to hand over this Fund to its custody on the ground that it was desirable to maintain it as a separate organisation for the benefit of the special class for which it was established. We congratulate the committee upon the wise decision at which it has arrived, and wish it many years' prosperity and useful work.

### NEWTON ABBOT.

The serious charges brought by a nurse against the management of the Newton Abbot Workhouse have been recorded in the form of an affidavit and forwarded to the Local Government Board with a request for an immediate inquiry into the matter. As it is ten or twelve weeks since these important questions were first discussed, it is most desirable that no avoidable delay should take place, inmates who have offered to give evidence having already gone out and others will probably soon be ready to be discharged. For all concerned it is essential that the truth or untruth of the various charges should be authoritatively decided.

### DISTRICT NURSING IN GLOUCESTER.

The Gloucester District Nursing Society appears to have done a vast amount of good work during its nine years of existence, and, moreover, it has done this so quietly that several members of the Board of Guardians owned, at a recent meeting, that they knew very little indeed about it. However, those familiar with the work of the nurses spoke with much appreciation of the value thereof, reference being specially made to over sixty cases which had been attended at the instance of the relieving officers—which must naturally effect a considerable saving to the poor rates.

### NURSES AT BISHOP AUCKLAND.

The Bishop Auckland District Nursing Association had its annual meeting last month, and it was proposed by the Rev. E. Price, the Rural Dean, and seconded by the Rev. Dr. Balgarnie, that there should be a stronger representation of working men on the executive, to widen the interest in the association. The work of the two lady nurses has been cordially appreciated by the sick poor, whom they have tended in their own homes.

### BETTER PROSPECTS FOR PAUPERS.

Last June The Hospital commented on the sadly neglected condition of the sick at Wolverhampton Workhouse, as described at that date in the able report of Dr. Totherick. In February of the present year attention was again called to the matter, Dr. Totherick, Mrs. Hatton, and the majority of the guardians showing themselves most desirous of securing adequate nursing for the pauper patients. Recently a letter has been addresed to the guardians by the Local Government Board, stating the lowest number of nurses which in their opinion could properly undertake the work. It is to be hoped that this recommendation for the considerable augmentation of the staff will receive the prompt attention which it merits. The Taunton District Nursing Association has made excellent progress during the last twelve months. It started very modestly two years ago, and now two trained nurses, a maternity nurse, and a probationer are constantly employed. A Samaritan Fund has been organised "to grant relief to absolutely necessitous cases without encroaching on the general funds of the Association." The nurses are kindly permitted by Messrs. Perkins and Saunders to use their omnibuses free of charge, a kind of privilege always appreciated by busy district workers, who need to save their time and strength in every possible way.

### A DANGEROUS PRECEDENT.

Old fashioned copy books made the little folks who struggled to emulate the beautiful head-lines familiar with many a wise and many a weak saying too. "Novelty is charming" was one of the fine "round hand" copies conveying but little meaning to the small and anxious scholar. In the light of later experience some people, however, would rather declare that "Novelty is dangerous," being fearful that innovations may be construed into precedents. For example when the Vicar of Greasbro' lately suggested to the Rotherham Board of Guardians that they should make a grant towards his parish nurse, "a conversation ensued in which an opinion was expressed that it would be a dangerous precedent," reports the *Sheffield Independent*. Yet since October 3rd this nurse has attended nineteen parish cases and paid four hundred and eight visits to them. She was also valuable during the typhoid epidemic. In the face of such services it seems as if the request of the Vicar for a subscription to the parish nurse fund were but natural, and certainly one which the guardians might comply with on the ground of economy to the ratepayers as well as in acknowledgment of services already received.

### A NEW NURSES' HOME.

The Jane Orr Memorial Home, in connection with the District Nursing Society, was recently opened at Ballymena. It has been erected by the inhabitants in memory of the lady who inaugurated trained nursing in the district, and who died in 1891 of typhus fever contracted during her work amongst the sick poor. The site for the new Home was given by Sir Hugh G. Adair, Bart., and the building is fully equipped with modern comforts and conveniences.

### SHORT ITEMS.

Mrs. Emily Bowden has been appointed nurse at South Molton Workhouse.—The Redruth Nursing Guild has been organised, and the members assert, according to the local press, that they have no desire to take the place of a trained district nurse, but "to carry out nursing work among sick poor as shall be deemed desirable."—Mrs. Mary Russell has been appointed fever hospital nurse of the Creom Union, at a salary of £15 per annum.—The annual report of the Bicester Nursing Home shows that the year closed with a small balance on the right side, which the committee will doubtless be glad to see increased in the future, especially as the work of the nurses appears to be thoroughly appreciated.—At the annual meeting of the Sunderland Nursing Institute Dr. Morgan is reported as having moved the confirmation of a resolution by the committee that in future the probation of nurses in the institute should extend over two years instead of one.—A nursing association for Linlithgow and district has been formed.—*The Idler* for April contains a very pleasant story by Dr. Conan Doyle, in which the characters of two doctors of opposite sex are admirably drawn.—There are seven nurses in training at the Lady Kinnaird Hospital, Lucknow.—Miss Stewart, Matron of St. Bartholomew's Hospital Lectured on Nurses' Recreation and Work, on March 30th, at 3, Hanover Square.

### A WOMAN'S NOTION OF "A FINE"!

A commission having been paid by a certain insurance company upon the premiums paid by nurses who have taken out policies in that company through the instrumentality of the Record Press (Limited), the proprietors of the *Nursing Record*, it is instructive to note a correspondence in *The Lady*. Thus, the Editor of the *Nursing Record* in a recent issue of *The Lady*, writes: "After a considerable experience of hospitals, we have only heard of one institution which fines its nurses, because thereat every probationer is compelled to subscribe to a certain insurance office, whether she requires an annuity for her old age or not." This is about the oddest definition of a fine which has ever been brought to our notice. Besides, we gather from the *Nursing Record* that the "one institution" is Guy's Hospital, and the "certain insurance office" is the Royal National Pension Fund for Nurses, a mutual society established for the benefit of Nurses. The best managed hospitals, realising that they have responsibilities and duties towards their nurses, are now helping them in the best possible way to make provision for the future. Whilst a few institutions have already their own special pension funds, many others find it more advantageous and easy to federate with that well-established insurance office, the Royal National Pension Fund. Whatever form of "insurance" against a destitute old age or cases of accident or sickness these pensions may take, hospitals are generally willing to assist in paying or adding to the premiums of their nurses. In fact, they "help those who help themselves," which is surely the most valuable aid which can ever be vouchsafed to independent womenkind. Can practical encouragement of prudent thrift be truly called "fines" by any effort of the imagination? Such a misnomer can hardly commend itself to those sensible, thoughtful women who have to provide for their own future as well as present maintenance. The dictionary gives "fine" as "a sum of money imposed by way of penalty for an offence," whereas in the Guy's scheme the payment by the nurse, so far from being "a fine," is in fact a premium for insurance of allowances in sickness and old age, which payment is augmented by the hospital by a bonus equivalent to more than 150 per cent. Nor is this all, for the premiums paid by nurses may be withdrawn by them at pleasure on leaving Guy's, with compound interest. The "fine," therefore, exists only in the fine imagination of the editor of the *Nursing Record*.

### TRAINING AT PENNSYLVANIA.

At the suggestion of Dr. Billings, Director of the Hospital and Training School of the University of Pennsylvania, the Board of Managers have decided that the course of instruction for nurses shall extend over a period of three years. In connection with the nurse training school a Nurses' Alumnæ and Beneficial Association has been organised "to promote good fellowship among graduates, and to establish a fund for their benefit in times of sickness or death." In the course of their training the pupil nurses are taught not only hospital but private nursing, the latter in the numerous small wards attached to the institution. Candidates for training are admitted from twenty-three to thirty-five years of age and are paid eight dollars per month during the first, ten dollars per month during the second, and eighteen dollars per month during the third year. This is to cover the cost of uniform, text books, and other expenses connected with the school, and is not looked upon as a salary. "The instruction given is considered a full equivalent for services."

### On General Nursing.

By Rowland Humphreys, M.R.C.S., L.R.C.P.Lond.

### VI.—CATARRH.

A catarrh is a "disorder primarily due to the action of wet or cold under unfavourable circumstances." It is a form of inflammation, which may be said to be peculiar to mucous membranes; it is not accompanied by fibrinous exudation, except in the more severe forms, nor is it usually accompanied by ulceration. It lines the organs of digestion, and the tubes leading to or connected with them. It also lines the urinary and genital organs.

### The Mucous Membrane.

Where it lines the mouth and nose and covers the lips it is readily open to inspection. Here it will be seen to be smooth, bright red, and to the touch it is moist and slimy from the mucous glands which are imbedded in it and open on its surface. It looks much the same in whatever part of the body it is seen, but its structure varies according to the special use of that part. It is necessary to describe shortly the microscopical structure of the mucous membrane, as the various parts of it will have to be referred to.

In all parts of the body it has a surface formed by the epithelium, made up of several layers of cells, which rest on loose tissue containing mucous glands, blood-vessels, nerves, and muscular fibres; the latter may be in one or two layers, and beneath it comes the sub-mucous coat containing vessels, nerves, and muscular fibres. Throughout all parts are scattered elastic fibres, and in the upper respiratory passages the epithelium has ciliæ on it. These ciliæ are moving processes which constantly move dust and such like material which has been inhaled in the direction of the nose. Catarrh may best be described under two headings—"The Acute Form" and "The Chronic Form," each of these having its own special effects on the part affected.

### Acute Catarrh.

The commonest form in which this is seen is that of coryza, or "a common head cold." This comes on soon after "taking a chill." Here it may be remarked that while a chill may cause a coryza, it may also cause any other acute affection; on the other hand, a coryza may be caused by any irritating substance, such as the inhalation of powdered ipecacuanha. A chill and a cold are by no means the same thing.

A sense of chilliness is experienced at the moment one "catches a cold," and whether the part affected be the limbs, body, head, or face, the impression produced passes by the sensory nerves to the brain, and there, if there is a spot specially liable to cold, the sensation is transferred to the nerves leading to that part, and in proportion to the condition and vulnerability of the part, so is the influence there produced.

It is not known what the nature of the change produced in the part actually is, but the appearance under these circumstances when produced artificially is that the mucous membrane becomes first of all very pale, and then deeply congested; in other words, the blood vessels of the part are first of all contracted and then greatly dilated. The result of this probably is that the membrane at the affected point is weakened and becomes susceptible to some morbid influence, probably of the nature of a germ, which has previously placed itself there, and which then obtains an influence or produces an effect such that in the course of a few hours the signs of a cold come on, and in a few days become well marked.

The patient at first experiences a fulness about the nose and a heaviness in the head, with a general feeling of chilliness and indisposition; these are followed by pain in the forehead and much sneezing.

### Stage of Hyperæmia.

The local symptoms are due to a dilatation of the little capillary vessels of the mucous membrane, followed by exudation of serum and a few leucocytes, the white blood-corpuscles, into the membrane. These result in swelling of the membrane, perhaps small hæmorrhages into it, a more or less complete blocking up of the respiratory passages, and an almost complete cessation of the usual secretion of mucus. The local effects of the cold usually begin in one small area, and spreading by continuity of tissue, may pass up the tear ducts to the eyes, and backwards through the back of the nostrils to the pharynx, æsophagus, stomach, and intestines, or from the pharynx to the larynx, trachea, bronchi, and lungs. The term coryza is applied more especially to the acute form of inflammation which is principally confined to the upper air passages, at any rate, at first.

### Stage of Watery Discharge.

The stage of hyperæmia already described passes on to that of free watery discharge from eyes and nose, which from its acrid nature irritates the nostrils and eyelids. The temperature, respiration, and pulse are all increased, the skin is dry, or may perspire freely. The nose is usually affected on one side to begin with, the inflammation spreading to the other side; it also travels up into the cavities between the layers of the frontal bone, called the frontal sinuses, and may pass into the cavity of the cheek bone, causing severe pain in these several parts, and may attack the throat, which becomes tender and aches. With the free discharge the swelling of the mucous membrane decreases, and the breathing becomes easier, and perhaps the patient may be able to breathe again through the nostrils. This stage is, then, characterised by the watery discharge and more or less severe general symptoms. It is very doubtful whether the symptoms can be entirely accounted for by the local irritation, as giddiness and other brain symptoms point rather to some morbid material circulating in the blood, which gives rise to them. Some of these can no doubt be accounted for by slight congestion of the liver which may even cause hæmorrhoids, others are probably due to the absorption of some poison from the irritated mucous membrane.

### Other Symptoms.

During this second stage the deeper parts of the mucous membrane become swollen with fluid, and white blood-corpuscles exuded from the capillary blood vessels. This all takes place within the first twenty-four hours or so. The mucous glands secrete mucous freely, at first of a clear jelly-like appearance, later it becomes very watery, containing much serum, and later still undergoing other changes and containing more leucocytes and epithelial cells. It becomes muco purulent and very thick. With the watery flow the pain and other symptoms undergo alleviation, but as the discharge again becomes thick the pain in the forehead increases towards night, and the pain in swallowing is combined with loss of taste, smell, swelling of the gums, and toothache from the periosteum of the jaws becoming affected, and there is often some amount of deafness, loss of voice, and tightness of the chest from implication of the various parts concerned, and if the catarrh extends to the stomach, changes similar to those already described take place, vomiting, diarrhœa, pain, and indigestion being the special symptoms, and if the catarrh affects the bile duct, by extending up it from its termination in the duodenum, some amount of jaundice, flatulent distension, &c., will occur, one special function of the bile being to act as an intestinal antiseptic.

A catarrh like this, if treated wisely, runs its course in ten days or a fortnight, if the patient is fairly healthy; otherwise, it may leave certain tendencies to other diseases, but usually the discharge becomes less and less, and thicker and thicker, the appetite returns, the expectoration looser, the cough easier, the epithelium, which has stripped off, reforms, the exudation is reabsorbed, and only an occasional rale remains to tell there has been any lung affection.

### Eastertide in Spain.

By a Nurse.

Passing from England, through modern France, into Spain, brought me most unexpectedly into contact with a bit of old-world life with its traditions and customs, and I cannot resist telling the readers of The Hospital a little of what passes in Madrid just before Easter.

On Palm Sunday not only do the churches present a picturesque appearance from quantities of green branches eight or ten feet long, carried by the various brotherhoods, but each house is adorned with a branch, great or small. The general effect of all this greenery is very charming, but there is something a little comical in the appearance of the mules which draw the tramcars being similarly adorned.

On Holy Thursday and Good Friday no carriages, carts, or trams are allowed, with the exception of those used by persons obliged to be "on duty." These carry special permits, and include the letter carts, a few station omnibuses, doctors' carriages, and so on. The centre of Madrid is, therefore, entirely free from vehicles, everybody going on foot, and mostly wearing black. All who can, wear new clothes. There is a Spanish proverb to the effect, "Who does not don new clothes on Holy Thursday cannot don them all the year." The women all wear mantillas with bouquets of violets or carnations fastened on their breasts.

On Holy Thursday the Queen of Spain always washes and kisses the feet of twelve poor women. The ceremony takes place in the Palace, in the presence of the Court, the Diplomatic Corps, &c. Then the stockings and shoes are replaced by the Queen's ladies.

An abundant fish dinner is provided for the poor women, and also for twelve poor men. The repast consists of twenty-four dishes, and the guests only go through the form of tasting them, when they are offered to them by the Queen herself, to whom they are handed by the Court ladies.

The dishes, which are almost untouched, are at once placed in large baskets, and most of them are sold to wealthy persons, who give them to charitable institutions, whilst the rest are purchased as curiosities by people, who willingly pay £2 or £3 apiece for them. There is also an allowance of about eight quarts of wine and some spirits to each poor person. The latter receive a goodly supply of new clothes, food, and money to take home with them.

The men and women are selected by lottery from hundreds of halt, blind, and maim applicants.

The Queen and her ladies are dressed in gorgeous raiment for this ceremony, with magnificent jewellery, all the Court being in full dress.

The custom dates from 1651, and if from any cause it has to be omitted, each poor person gets an ounce of gold (about £3) instead. Admission to witness this ceremony is by tickets.

In the Cathedral on Holy Thursday the Bishop washes the feet of young men who are about to be ordained.

On Good Friday another interesting ceremony takes place in the Palace at Madrid, all present wearing mourning. The Bishop, who presides at the service, at a certain point hands to the Queen a parcel of documents tied up with black ribbon, asking: "Will you pardon these that God may pardon them?" And, in a trembling voice, the Queen makes reply in the affirmative. Then the Bishop immediately undoes the black ribbon which bound the papers and replaces it by a white one. These documents relate to the cases of criminals condemned to death, and every Good Friday, as well as on other occasions, such as the King's birthday, similar pardons are granted to a certain number of persons.

On Good Friday in the present year no less than sixteen were pardoned, some of them having been sentenced in 1891. Many were soldiers between the ages of twenty and twenty-five, condemned for military offences, others for horribl assassinations, chopping up old women, &c.

The reason of the pardons is, therefore, to an English woman somewhat of a mystery.

There are many processions both in the streets and churches on Good Friday, and numerous old statues are carried round presumably illustrative of the death and burial of Christ. In Seville they are said to be most imposing, people travelling from long distances to witness them; but in Madrid the street processions at any rate are far from imposing, in spite of the bareheaded officials and the crowds which accompany them.

Easter Day is chiefly remarkable as the date on which the bull fight season opens. All the women wear white mantillas. "A los toros" is yelled on every side, the galloping of mules and horses, the transit of vehicles of all imaginable sizes and shapes, the shouting and the coming and going of some 12,000 spectators form a brilliant spectacle, although it is difficult to realise in the streets of Madrid that Easter Day falls on Sunday!

### Women's Volunteer Medical Staff Corps.

A meeting to consider the steps to be taken with regard to the formation of the Women's Volunteer Medical Staff Corps was held on March 31st at the Ideal Club in Tottenham Court Road. Several speakers—among them Mrs. Heatherley (chairman), Miss Stokes (hon. secretary), and Dr. Alice Vickery—set forth the advantages to be obtained by the establishment of such a body, and also discussed the question of dress; the costume most in favour appearing to be a combination garment of vest and knickerbockers, over which a skirt could or could not be worn, as was most convenient. Major Sargent thought that women would hardly obtain recognition of their military rights from authorities who denied them civil rights; and he reminded the would-be members of the corps, who intend to go in for a course of musketry drill, that the Medical Staff Corps is a strictly non-combatant body. He thought that the energies of those who wished for work of the kind would be more usefully directed towards the St. John Ambulance Association; while he doubted whether the ladies who spoke of subjecting themselves to military discipline were aware of what that meant, and if they would at all relish twenty-eight days' cells, on bread and water, as punishment for a breach of duty. The general feeling of the meeting, however, appeared to be in favour of the proposed movement, which some forty ladies have already promised to join.

### Bedford.

### THE OPENING OF THE NURSES' HOME.

The Duke and Duchess of Bedford were present at the re-opening of the Trained Nurses' Institute at Bedford last week. Increased accommodation has been secured to the staff by the addition to the building of a new story, ingeniously contrived without removing the roof. The total cost of the alterations will probably be about £700, and £600 was raised by a bazaar last year organised by Mrs. Rawson, the Lady Superintendent, for this purpose, and various subscriptions from other friends have been received. The opening ceremony was largely attended, and a report of the work and its growth was read by Mr. Whitbread, M.P. There is now accommodation for a superintendent, 28 private, and one district nurse.

In the course of the proceedings, the Duchess of Bedford said that she had been asked to present a testimonial to Mrs. Rawson in acknowledgment of the zeal and capacity she had exercised during the last five years as Lady Superintendent of what had now become a permanent institution. The illuminated address which was handed to Mrs. Rawson was accompanied by a purse containing £60.

### Lectures at St. George's Hospital.

THE ANTISEPTIC SYSTEM—(*continued*).

Dressings.

There is an idea prevalent among some people that with antiseptic surgery dressings are everything; but they are not, they are but a detail, an important detail, it is true, but a part of the system and no more. The worst perhaps of the antiseptic system is that every detail is so important, almost in fact essential. On the perfection with which all details are carried out success depends, and success may mean the preservation of life, and want of success the death of the patient. Take a wound, especially a hollow wound, where a cavity will be formed after the skin has healed; here discharges are inclined to collect and thus form the best possible pabulum for injurious substances to enter. There is, of course, a great tendency for mischief to arise in any wound not protected from the air, and the whole business of dressings is to filter the air and so to protect the wound. Dressings are made of gauze, or some widely-meshed material, saturated with antiseptic agents, such as eucalyptol, carbolic, thymol, or corrosive sublimate. These are variously coloured, in order to be easily distinguishable. But the quantity of antiseptic solution thus absorbed is infinitely small, far too little to have any power to destroy germs already in a wound, though it may prevent their thriving and multiplying. Put the most beautiful antiseptic dressing in the world over a dirty wound and the fermentation will go on just the same inside. Wool is a most efficient filterer, and will arrest all injurious substances, but if ultimately the discharge soaks through the dressings, then you have a continuous track of pabulum through the dressing to the open air. If this happens and the dressings are at once examined microscopically, in the layers of dressing nearest the outside air numerous bacteria will be found, fewer and fewer in those nearer the wound. This is why it is so necessary to notice when the discharge is "through." If a dressing is not surgically clean it is absolutely useless antiseptically. Dressings, then, have a limited power, but if they have not a strong power for good, they have a very strong power for evil. It is too often imagined that because a dressing is coloured blue or pink it thereby confers immunity. But not so, for plain lint or anything else would do as well if it were not surgically clean. Dressings should be kept in air-tight boxes. In old days when antiseptic surgery was first practised it was said if this or that was not done the system was very imperfectly carried out. Mr. Dent spoke of one surgeon who was most particular about boiling his instruments, but this very man did not change his coat when he operated. He would rub his coat against a wound, and then stand up before an audience of people who knew what they were listening to and talk about the advantages of his perfect antiseptic surgery! However, this man did well in his profession, and is now no doubt still rubbing his coat against wounds.

Preparation of the Patient and Operation-room.

Great care must be taken to carefully cleanse the skin of the patient before an operation and round a wound, but laudable efforts in this direction must not be overdone, and the patient must be cleansed without being chilled too. A student was once asked to describe an operation on the head, and he gave a minute account of the antiseptic precautions to be taken beforehand, but not a ghost of an idea as to how the operation was to be performed! Due regard should be paid to the patient's comfort while preparing the skin. In sponging with ether it should be remembered that the temperature is lowered enormously, which would be sufficiently trying to a person in health, and how much more so to one who is ill? It should be remembered that in shaving the affected part a great deal of the sebaceous matter is got rid of, and dirt with it.

Only pure atmosphere should come in contact with a wound. Some people think that by starting a spray they cleanse the air of a room. It may do so, but it certainly makes it damp. There is a great tendency in private houses to get a room ready for an operation by thoroughly sweeping it, but where a room which is dirty has to be used for an operation, sweeping it on the morning of the day only makes mischief. It would be better to let quiescent dust rest. Air must come in and dust with it. The cleansing of the hands of all who come in contact with the wound is of great importance, and much care should be exercised in the matter of nail-brushes. The ordinary nail-brush is a regular germ-trap. Each person should have their own, and there should be one for use before the operation and one for after use. Bailey's rubber brushes are very good for this purpose.

Sponges are responsible for an immense amount of the trouble arising in wounds, and are most difficult to get and keep clean. Substitutes have been tried—cotton-wool plugs, &c.—but they are more expensive and not always satisfactory. A good artificial sponge is made by Borroughs and Wellcome, consisting of a ball of absorbent wool, in the centre of which is a little capsule containing some antiseptic agent, carbolic or eucalyptol, which is broken at the moment of using and the fluid distributed throughout the sponge. There is no doubt that sponges have been frequent carriers of infection. In old days there used to be certain "ward sponges" to the use of which many cases of erysipelas might be traced.

On the whole it may be said that the antiseptic system is the best that we have, but very imperfect as yet, and something better may be looked for as time goes on. Already there are signs of simplification, and we are using plain boiled water where it once was thought necessary to use carbolic; but if you give the patient a chill and he dies of pneumonia, it is of no use that you have prevented fermentation. The patient must be considered as a whole. One objection urged against the system is that it is expensive; and so it is. We have not yet learnt to be sufficiently economical, but on the other hand it unquestionably diminishes the amount of pain, of traumatic fever after operations, &c., and furthermore it saves life, and it will be time enough to talk of the drawbacks of the system from expense when the exact value of a human life has been determined. Unquestionably the antiseptic system has revolutionised surgery.

In conclusion Mr. Dent impressed upon nurses the enormous responsibility which they share equally with the surgeon in carrying out perfectly each and every detail of antiseptic surgery.

### Deaths from Carbolic Acid.

A return of the number of deaths caused by the taking of carbolic acid, distinguishing males and females, which has lately been printed by order of the House of Commons, presents two rather curious features. In the five years, 1887 to 1891 inclusive, there were altogether 375 deaths from this cause, of which 182 were of males and 193 of females. The first point is that of 138 accidental deaths the men numbered 84 and the women only 54, and in every single year shown in the return the men outnumbered the women. The second point is that in the case of 236 suicides this order was reversed, only 98 being men, while 138 were women. Although in two of the specified years more men than women took their life in this manner, yet in 1888, of 64 such suicides, the men numbered only 20, as compared with 44 women, and again in 1891 they were in the proportion of one to two, the actual figures being 21 males and 42 females. The only murder committed by this agency in the five years was committed by a woman. It is singular that such a particularly painful death should exercise a greater fascination over women than men, and it would be interesting to ascertain whether the fact has any other explanation than may be found in the caprice of a morbid mind.

### The Mutual Relations of Nurses and Medical Women.

By Mrs. Scharlieb, M.D., B.S.

I should much like (through the Editor's courtesy) to ventilate a question in which I have long been interested, and which forms the subject of a communication in a recent issue of The Hospital. In the paper entitled "Can Medical Women Nurse? By One Who Knows Them," the appointment of a medical woman to teach nursing to the girls attending a Board school is made the opportunity of going into the subject of the mutual relations of nurses and medical women. This is a question which has long been a matter of interest to me, and one in which a more thorough mutual knowledege and understanding cannot fail to be advantageous to all concerned.

May I say, before beginning the consideration of the subject proper, that my interest in nurses and in women doctors is of no recent date, for I began my professional work in the Government Lying-in Hospital, Madras, in 1872, and was for a short time Lady Superintendent of the General Hospital, Madras, in 1878. I then had to arrange for the conduct of the entire nursing establishment, including the training of the nurses and probationers. Since that time my work has brought me into intimate relations with many nurses, and has greatly enlarged my views of woman's work, and woman's preparation for that work, especially in reference to medicine.

The writer of the communication referred to brings forward several points which, in the mutual interests of nurses and medical women, should have further discussion. To summarise briefly, the points appear to be: (1) That women doctors are not the most suitable teachers of nursing. (2) That women medical students commence their studies too young. (3) That medical women students do not avail themselves as they might do—and as their brethren frequently do—of opportunities for "picking up hints on nursing." (4) "That women students are antagonistic to nurses, whilst trained nurses invariably cherish an instinctive aversion to the awkward, self-conscious young girl." (5) That medical "women should be before the other sex in courteous demeanour, but, alas! they are not so." In considering the first question, whether a medical woman is a suitable teacher of nursing, it is at once clear that medical women differ widely in their gifts and aptitudes, that some of them were trained nurses before they commenced their medical studies, that some of them have a strong natural aptitude for nursing, and that some of them have no such gift. The fact that a person possesses medical or surgical knowledge does not necessarily enable him or her to teach it, and the fact that a woman is a doctor neither makes her a nurse nor does it prevent her from being a nurse. Further, we must allow that the fact of being a trained nurse does not necessarily confer the gift of teaching. The fitness of anyone to teach nursing depends on two conditions: first, that the would-be teacher must know the subject to be taught; and secondly, that he or she should possess the faculty for imparting knowledge. Therefore the suitability of the selection of a medical woman to teach nursing depends entirely on the *personnel* of the woman selected. Other things being equal no doubt the person having the greatest knowledge and experience should make the best teacher and, therefore, a surgeon should teach surgery, a physician medicine, and a nurse nursing. We all know that art is long and life is brief, and no one can expect to be equally competent all round, hence the necessity for specialists. All the same, the general practitioner is as valuable and essential as the specialist, and it may well be that in some districts in England, and still more in distant lands, there may be no one with the elements of nursing knowledge except medical folk, whether men or women. In such case the doctor must train the most promising candidates, and that this can be well done is proved by the experience of the Government of India and by the history of the Dufferin Fund, nurses having been fully and successfully trained by both medical men and medical women.

2. That medical women students commence their work too young. "Unfortunately many young ladies take up their medical studies whilst still in their teens, half a dozen years earlier than they would be allowed to enter on the duties of a probationer nurse." The writer may well say unfortunately. It is very sad to see girls of eighteen who are necessarily inexperienced, and with imperfect ideas of life, entering on a career that must soon lead them to knowledge of good and evil. The arrangement has its very serious drawbacks, but is probably inevitable. The General Medical Council now requires that all medical practitioners shall have five years' training between registration as student and registration as practitioner. Thus a student commencing work at eighteen cannot be qualified until twenty-three, even if every examination is passed as quickly as possible; while, apart from laziness and stupidity, a casual illness or some other accident may easily postpone qualification until the age of twenty-five. Thus the girl who begins medical studies at eighteen and the girl who begins her probationer's work at twenty-three are fully qualified, each in her own branch of the profession, at about the same age. The young medical woman will then seldom at once enter upon independent practice. She generally joins a senior or works for a time under supervision. For those who can afford to wait it is far better not to commence medical studies at eighteen. Far better to take the B.A. or B.Sc. degree, to devote themselves to scientific cooking, dressmaking, or the duties of house-daughter—anything, indeed, to teach them self-reliance, self-control and patience. We have to remember that now when our girls are needing as expensive an education as our boys, that the fathers are few who can afford to support a family of sons and daughters until each attains the age of twenty-five or thirty. If girls are to be as well equipped for the battle of life as are boys, then all must bestir themselves, and not be needing too long the infantile method of feeding.

(*To be continued.*)

### Clapham School of Midwifery.

That the training should be most thorough in the special branch of work before them is a lesson emphatically impressed on all the students at the Clapham School of Midwifery. There are two centres of work—one at 131, Clapham Road, and the other at 18, Albert Road, Battersea—and at both the women students reside with the women doctors "in charge of the maternity work, and are thus constantly in touch with them regarding the management of individual cases." At Clapham the work lies both in the Clapham Maternity Hospital (which has 36 beds) and among out-patients. At Battersea out-patients only are attended, except by special arrangement. Dr. Annie McCall is director of both branches, and she does a particularly good work by drawing attention to the vital necessity for medical women aspiring to practice in India, being specially well prepared for their responsibilities, as they will there "at once find themselves face to face with the most difficult obstetric practice without the advantage of consultants."

### Wants and Workers.

Votes urgently needed for the Putney Incurable Hospital at the May election. It is the second election of this poor woman, and it is very desirable that she should succeed this time. For three years she has been attended by the Haggerston and Hoxton district nurses, and is now in a small Paying Home; but the money which was collected to put her there is almost exhausted. Votes will be gladly received by the Superintendent at the Nurses' Home, 105, Nichols Square, Hackney Road.

### The Nursing of Army Sisters.

BY A. O. GREY.

I.

ON leaving the hospital, where her time of training has been spent, the newly-fledged nurse naturally wishes to find out for what sphere in the nursing world she is best fitted, and which among the many pathways will lead her to congenial work and advancement.

One of the most popular appointments is that of Sister in the Army Nursing Staff, but usually there is little known in civil hospitals of the requirements, the disposition, and general fitness which go to make a successful sister. Even among matrons, to whom the probationer turns for advice, ideas on the subject are vaguo, and very often misleading. The general tendency is to underrate the work, and overrate the amusements and social pleasures that are supposed to predominate in military nursing. In reality there is actually more freedom during the hours off duty in the training school than there is for the Army Sister after she has passed her probation at Netley, and been sent to a station hospital, where the staff rarely numbers more than three. No one therefore should waste time waiting for a vacancy (and this period of waiting is usually considerable, for candidates are many, and vacancies few) who does not mean to work hard during the whole of her career, as senior and junior alike have an equal amount of ward duty allotted to them, and succeeding years only bring increasing responsibilities.

The probationer should, if possible, train in one of the general London hospitals ; Edinburgh and Liverpool are also excellent schools, and they also give the three years' certificate which is one of the essentials required by the Director General of the Medical Department, in whose hands the appointment of candidates rests. She should acquaint her matron with the object she has in view, so that she may have special medical and surgical training in male wards.

Another qualification is that the candidate, to quote from the regulations issued with Army orders, "must produce a recommendation from some person of social position—not a member of her own family—to the effect that her family is one of respectability and good standing in society "—in other words, she must be a lady. It will be found that nursing in civil and military hospitals is widely different. In the former there is a sister in whom discipline is vested, staff nurse, and one or more fellow probationers. The patients are well accustomed to petticoat - government. But in the latter, the sister is in charge of a division or block, and has no other woman with whom to consult or converse, except during the occasional rounds of the lady superintendent. A ward-master, who is a non-commissioned officer of the Medical Staff Corps, is responsible for the discipline and cleanliness of the wards, but it is from the bearing of the sister that the ward takes its tone. She goes round with the surgeons and receives their instructions, either carrying them out herself, or seeing that the orderlies do so. It is a matter for discretion how much may be safely trusted to these subordinates, and here it may be well to remark on the positson which the sister occupies in regard to the orderlies.

The sister who is interested in her work and patients would prefer to do most of the poulticing, antiseptic dressings, &c., herself, but there is a duty to the orderlies to be thought of. They are trained theoretically in nursing at their depot, Aldershot; but as they are for the most part lads who join the M.S.C. on account of the extra pay, or because they are physically unfitted for any other regiments, the love of real nursing is frequently absent, and their duties are performed in a very perfunctory and hap-hazard manner. Their practical training is to be completed in the hospitals to which they are drafted, so that if the sister carries out all the treatment herself, the orderlies cannot become the practical nurses they ought to be to undertake the entire care of cases in the smaller stations where there are no sisters.

An instruction-class is held daily, when the surgeon, and afterwards the sister, give "lessons" for a stated time, but probably instead of being interested and anxious to learn, they are longing for the hour of release to come, so that they may practice for the next cricket or football match. Example is ever better than precept, and if possible the sister should have the assistance of her class in carrying out the surgeon's orders, so that practice may make perfect in bed-making, the care of helpless patients, &c. This, however, is not always easy to manage. More than likely, when the morning's work is in process, the orderlies are required for drill, parade, or fatigues. Great tact and patience are therefore required to make these men efficient nurses; their answers and reports are given with much glibness—until one knows them, it seems almost incredible that things are not as they appear. It is most difficult to make an orderly understand that if stimulant is ordered in drachm doses every half-hour, the double quantity every hour, or even greater interval, would not answer the same purpose ; and this is only one instance out of many of the difficulties the sister has to contend with in teaching the young idea how to administer nourishment or medicine.

Gifted with refinement, tact and good temper, combined with firmness and common sense, there will be little doubt that an Army Sister will have a most happy and prosperous career, and be thoroughly fitted for the duties and position she takes up.

### Nursing in the United States.

OUR NEW YORK LETTER.

THE constitution of the Superintendents' Society is as yet very dry bones, and is almost a copy of the Superintendents' Society of Medico-Physicology, as it is called. Dr. Hurd furnished Miss Hampton with the skeleton, and it remains but a skeleton as yet.

The Commissioners, on the recommendation of the Medical Board of the New York Hospital, have placed the superintendence of the Male Training School in the hands of Miss Louise Darche, already the head of the female nursing department of the hospital. This addition gives her the responsibility of twenty-two young men and one head orderly. Formerly there were two head orderlies, but the senior has now been supplanted by a supervising nurse, a graduate of the school. She will have the immediate charge under the Lady Superintendent.

Miss Darche is not very sanguine over the success of this experiment, but means to give it a fair trial, and if it fails, then female nurses will be introduced. Miss Darche's well-known organising ability leads people to hope, however, that she will solve the difficult problem by proving that an adequate amount of training can make suitable men into efficient attendants on the sick of their own sex.

The times have been so hard in New York this last winter and the season so healthy that private nursing sufficient to keep the graduate nurses busy has been hard to get.

Small-pox broke out in the New York City Hospital during the winter, and as this happened in a dermatological ward, was not suspected until about a dozen persons became infected, the warden amongst others. Consequently the hospital has been more or less in a state of quarantine. Cook's County Hospital and several other large ones have also been infected with small-pox this winter.

The book of international papers read at Chicago has come out in very good form, and is doubtless already in the hands of many HOSPITAL readers, as it is published in England.

### Women's Work.

PRACTICAL MASSAGE—(*continued*).

By a Masseuse.

Therefore to be a successful masseuse it is well that a girl should be of a discreet age, but at the same time not too old, for although a woman of fifty may be as good a rubber as one of thirty, yet it is not likely that she possesses the same amount of health, vigour, and electricity. Half the benefit accruing from massage is the electricity which the masseuse gives out. Some say that a masseuse speedily becomes old and worn out because she bestows so much of her own strength upon others. At any rate, those who would become masseuses must have good healthy constitutions. I purposely omit the word strong, for I feel convinced that if the patient receives benefit so does the masseuse. If she is not robust to begin with she very soon becomes something akin to it.

Massage and nursing go hand in hand—indeed, almost too much so for the girl-labourers who are daily coming forward to take up the former only. They find, however, that nursing and massage are being combined more each day. Had the two things been kept apart, I think there would have been ample room for all.

It may not be out of place, perhaps, if I give a few practical hints as to the cost of learning massage. Of course, this varies according to the teacher and school; the usual fees run, however, from five to ten guineas—not for so many lessons, but for instruction till perfected. It is not too much when one considers that a profession is thereby acquired. It is the custom with most teachers to make a reduction in their fees as regards trained nurses. The "period of learning" or number of lessons required before a pupil can be said to attain proficiency in manipulation depends greatly upon the pupil herself. If she be apt and clever, able to take in readily the instruction she receives, with hands prompt to obey, and capable of gliding with a certain amount of facility over the different parts of the body, she may, according to some methods, be deemed proficient in three weeks' time, or even a fortnight, and granted a certificate of eligibility and merit; but many persons take two months, or even longer, before they obtain sufficient skill or ease with their hands to render them desirable masseuses.

And here let me enforce the desirability of learning upon a living subject; it is an utter impossibility, not to say actual absurdity, to imagine for one instant that a woman can turn into a qualified rubber without having constantly practised as a probationer upon the human body.

After a certain number of lessons should a pupil continually fail to remember the various movements and display a want of confidence with regard to herself, she had better give it up at once—she will make nothing of massage. As a general rule, however, if a girl goes in steadily for the work for a month or so, studying meanwhile one or more of the leading books on massage so as to master the art not only with her hands, but her head also, and if she attends lectures on anatomy, as a rule she will find herself at the end of this period a certified and eligible masseuse.

A young masseuse will not find cases drop into her hands; she will have to do her best and work hard to obtain patients, for as in every other branch of woman's work the market is over stocked. Neither gold nor patients are obtained without trouble. Sometimes success is achieved early by chance or influence, or more often through the recommendation of friends or doctors.

If a masseuse possesses no friends able to keep her, or no doctor allies, the question forces itself disagreeably forward, "What is to be done?" Advertise? Yes, that is not at all a bad plan, and often answers in the long run. Circulating cards are also not to be despised, a chance patient is often obtained in that way. But I think, if possible, the best thing of all is to try and get into some establishment, hydropathic or otherwise, where masseuses are employed, for a few months at any rate. There a masseuse will meet with all classes of patients and ailments, and gather experience, testimonials, and recommendations, which will serve her in good stead when she sits up later on her own account.

A young worker, however well taught and efficient, cannot expect to command the same fee that an older and more experienced rubber would demand, as a matter of course. She may think herself fortunate if she receives at first five-and-sixpence a rubbing, or from thirty shillings to two guineas a week. It depends greatly upon what is required; attendance twice a day, with the use of the battery, deserves a higher remuneration. I have known masseuses receive as much as four guineas weekly from one patient, but such cases are rare. Taking the good with the bad, a masseuse when once she becomes known, and has managed to make a small connection of her own, earns a fair livelihood on the whole, taking on an average three or four pounds a week—sometimes more, sometimes less. At the present moment times are bad in the massing world, as indeed everywhere. Let us hope there will soon be a change for the better.

### Where to Go.

St. Martin's Hall, Charing Cross.—On Friday, April 27th, at three p.m., annual meeting of the Zenana Bible and Medical Mission.

Trained Nurses' Club, 12, Buckingham Street, Strand.—Dr. Savage will lecture on Friday, April 13th, at a quarter to eight. Subject: "Epilepsy and the Convulsions Associated with Childbirth." A few tickets to non-members at 6d. each to be obtained from the Secretary.

Queen's Hall, People's Palace, Mile End Road.—On Tuesday, April 10th, at eight p.m, a grand ballad concert, under the patronage of Mr. Spencer Charrington, M.P., will be given in aid of the North Eastern Hospital for Children, Hackney Road. Tickets, 2s., 1s., and 6d.

Morley Halls, 316, Regent Street.—On April 25th and 26th the annual conference of the Young Women's Christian Association will take place. The meetings will begin at half-past ten. Particulars can be obtained from the hon. secs., the Hon. E. Kinniard, 115, Mount Street, and Miss Morley, 47, Grosvenor Street.

### Notes and Queries.

The contents of the Editor's Letter-box have now reached such unwieldy proportions that it has become necessary to establish a hard and fast rule regarding Answers to Correspondents. In future, all questions requiring replies will continue to be answered in this column without any fee. If an answer is required by letter, a fee of half-a-crown must be enclosed with the note containing the enquiry. We are always pleased to help our numerous correspondents to the fullest extent, and we can trust them to sympathise in the overwhelming amount of writing which makes the new rules a necessity. Every communication must be accompanied by the writer's name and address, otherwise it will receive no attention.

### Queries.

(33) *Queen's Nurses.*—Can you give me any information about the Queen's Nurses, especially as to how to proceed to be trained as one?—*Anne S.*

(34) *Male Nurses.*—Please give name of any hospital where men are trained?—*H. H.*

(35) *Monthly.*—Is it possible to earn a living by monthly nursing alone, and where can it be learnt free?—*Kate.*

(36) *District.*—Where can I get rules, &c., suited for a district nursing association?—*Nurse Amy.*

### Answers.

(33) *Queen's Nurses* (*Anne S.*).—Apply to Miss Peter at St. Katherine's Hospital, Regent's Park, for all particulars. Regarding your other questions, please read notice just above.

(34) *Male Nurses* (*H. H.*)—No English general hospitals undertake the training you require. You get a list of asylums in "Burdett's Hospital Annual," pub ished by Scientific Press, 428, Strand, London.

(35) *Monthly* (*Kate*).—The Secretary of the Midwives and Trained Nurses' Club, 12, Buckingham Street, Strand, will give you all particulars.

(36) *District.*—Write to Miss Peter, Queen Victoria Jubilee Institute, St. Katherine's Hospital, Regent's Park; or to Miss Hughes, Metropolitan and National Nursing Association, Bloomsbury Square.

☞ For Everybody's Opinion, Reading to the Sick, Book World for Women and Nurses, &c., see page ix. et seq.

### The Muses' Looking Glass.

### WHERE TO SPEND A HOLIDAY—ALONG THE ROMAN WALL.

For anyone who is capable of more of a walk than a two or three miles saunter, few places present more attractions for a "busily-idle" week than the neighbourhood of the Roman wall.

To south country people the Roman Wall is little more than a shadowy name associated with the history-books of childhood. We never for a moment imagine that any parts of that grand national barrier still exist, but they do, and the tourist need not necessarily be much of an antiquary to understand and appreciate the grand masses of hewn and chiselled masonry, the camps, the guard-houses, the fosses and vallums, of which there is abundant evidence at intervals from Carlisle on the west to Newcastle on the east.

Originally built by Hadrian, A.D. 120, the wall was repaired and extended by Severus about one hundred years later, and served to protect the Romans and the conquered Britons from the Picts and Scots.

The stones of which it is built are mostly whinstone and granite, all carefully shaped, and in many instances brought from a considerable distance, for the Romans as usual showed their thoroughness in not taking what lay nearest unless it was exactly suitable.

The wall was originally 18 to 20 feet high and eight feet broad, two chariots being able to drive on it abreast. General Wade in whose honour was made the historic couplet about the Scottish roads,

"If you'd seen these roads before they were made
You'd lift up your hands and bless General Wade."

appears to have used up an immense quantity of wall stones in order to construct the road from Newcastle to Carlisle, and it is along his road the tourist walks, having England on the one hand and miles of Debateable Land on the other, and a very glorious expanse of country it is, wild and boggy for the most part, with no sign of a human being or of his habitation as far as the eye can reach; altogether a most restful prospect for a tired worker's weary eyes, the absolute quietness and utter absence of movement constituting in themselves pure enjoyment.

It does not require a very vivid imagination to picture parties of mosstroopers and cattlelifters wending their way over the moors among the heather. What a fine point of vantage for keeping these gentry in order "Belted Will" had in the neighbouring castle of Naworth, when he was Lord Warden of the Marches.

Gilsland, about forty minutes by train from Carlisle, is a good centre to stay at, from it the Nine Nicks can be easily reached on the one hand, and on the other is Burnt Oswald, which has the remains of a particularly fine camp with guard-houses, remains of villas, and of a hot air bath, all being enclosed by the original wall. On the stones of the floor of the guardhouse gateways, the marks that the chariot wheels have furrowed into the stones are still plainly seen; here also several stone altars with inscriptions, and figures have been found, and are now preserved in the farmhouse which is built on one side of the camp, with the wall stones. Roman remains consisting of rings and intaglios, stone figures, household and temple altars, and flat incised stones have been found in plenty all along the neighbourhood of the wall, and nearly all of them in a wonderfully good state of preservation.

After walking over the sixty miles along which the wall originally extended, or at all events over as much of it as the holiday-maker feels inclined for, Naworth Castle and Lanercost Priory must be seen. Both of these stand in a valley on the English side of the wall, and are separated from each other by a road and delightfully brown peaty coloured river, which is crossed by means of rather perilous stepping stones.

Naworth belongs to Lord Carlisle, who allows it to be inspected at all times, Lady Carlisle, in fact, carrying her pet hobby of socialism so far as to beg the tourist "Not to mind their being at lunch, but to come and look round at everything," and so though somewhat abashed at intruding, the tourist does look round, and sees a magnificent specimen of an old Border fortress castle turned into a modern dwelling, the transformation having been effected with such skill and good taste that the modern books and pictures and the old wood-work, the tapestry, and the Burne Jones' studies, the Florentine inlaid cabinets, and the big locks and little peep-holes through the panels into the courtyard, all seem to fit into a most harmonious whole.

Belted Will's Tower and the Dacre Tower still remain very much as they were originally, and in the former, Belted Will's bed with its costly hangings, his library of huge old books, his chapel, and his chairs and tables are all supposed to remain as he left them. He was by birth a Howard, being the third son of the unfortunate Duke of Norfolk who was beheaded by Queen Elizabeth in 1577, and he married his half sister, Lady Elizabeth Dacre, the heiress of the lands and castle. It was in the somewhat arbitrary exercise of his power as Lord Warden of the Marches, that he acquired his nickname, for, let the marauders be ever so fleet, "belted" Will was always ready booted and spurred to ride after them, and when caught, tradition says that they were hanged on a neighbouring oak tree with a conveniently overhanging bough, the tree as it still stands being decidedly suggestive of a ready-made gibbet.

But this is an age of whitewash, and the border hero is now made to pose as a peaceful literary man—witness his books; and as a deeply religious one—witness his oratory; but the tourist is inclined to hark back to the original idea when he sees at one corner of the oratory a little cupboard-like door in the wainscot and finds that through this door (made sufficiently large by lifting up a plank in the floor) the old hero, when nominally at his private devotions, was accustomed to descend into the deep dungeons below by means of a secret stair in order to torture and question his prisoners!

The dwelling-rooms are now mostly on the second storey running round the quadrangle and from these rooms several somewhat breakneck and dark spiral stone stairs lead into the offices and the garden. It must be a fine place for ghosts!

### The New York Teachers' College.

The Teachers' College in New York is one of the school forming a university system which has in the course of the last four years become a well-established and most important feature in the education of the rising generation. Far from believing that teachers "are born, not made," our American contemporaries do their best to show how perfect a scheme can be evolved for the manufacture, so to speak, of fully-equipped instructors and instructresses.

The demand for teachers trained at the New York school so far exceeds the supply that it has been found absolutely necessary to increase the accommodation, and thus to enable the trustees to admit a greater number of students. The site on which the new buildings are already in course of erection is a piece of land with a fine view of the Hudson, the Harlem, &c. The land was a free gift from Mr. George W. Vanderbilt, and the cost of the edifice will also be defrayed by various friends of the movement.

As regards the methods followed in the actual education of the pupils, we recommend those of our readers who are interested in the matter to read an admirably illustrated article in the *Cosmopolitan* for March by Rosa Belle Holt, one of The Hospital contributors.

### Everybody's Opinion.

Correspondence on all subjects is invited, but we cannot in any way be responsible for the opinions expressed by our correspondents. No communications can be entertained if the name and address of the correspondent is not given, or unless one side of the paper only be written on.]

A Trained Nurse Who Speaks from Experience writes: It was with great interest that I read in The Hospital of December 2nd, 1893, the comments on the Royal Hospital for Incurables, Putney. Knowing the great interest you take in nursing and nurses, I wish to bring to your notice a few of the defects in that branch of the work at Putney. It has been suggested that all the nurses should be hospital-trained, but great reforms would be needed for the comfort and accommodation of a nursing staff, if the committee wished to retain their services when appointed. The present bedrooms occupied by the assistant nurses and wardmaids together, are a disgrace to any public institution. Most of the bedrooms are in the basement of the hospital adjoining coal cellars, stores, heating apparatus. &c.; each room contains four, five, and some even six beds, without either screens or curtains to divide one bed from another. Then the common mess-room, used for nurses, servants and wardmaids, contains the sink, where most of the washing-up is done for the staff, there being no pantry for that purpose. A huge fire is kept on the hottest day in summer, as the tea, coffee, &c., required are prepared in this mess-room. The table requisites leave much to be desired, and I hardly think one of the committee would offer those at present provided to their own servants.

### GENERAL TRAINING.

"A Monthly Nurse" writes: I shall be very glad if anyone can tell me of a provincial hospital where a few months' training in general nursing can be obtained at reduced fees by a monthly nurse?

### PRIVATE NURSING ABROAD.

"Rabbi" writes: Can anyone give reliable information to a nurse respecting the prospects open to one starting on her own account at Durban, Johannesberg, Cape Town, or Port Elizabeth?

### NURSES IN CEYLON.

The Hon. Secretary of the Ceylon Nursing Association writes: There is work out here for at least half a dozen more nurses. They must have had experience in private work, and be qualified midwives in addition to their hospital training. The association is willing to pay half their passage mon ey,and they would rarely be out of work.

### Appointments.

North Herts and South Beds Infirmary, Hitchen.—Miss Grace Hall has been appointed Matron of this infirmary. She was trained at the Derbyshire Royal Infirmary, and had charge of wards there. Miss Hall was afterwards on the private nursing staff of the West Kent Hospital at Maidstone, and for nearly three years night superintendent at the Hull Royal Infirmary. Her testimonials are excellent, and we wish her every success.

### Minor Appointments.

Brentwood Cottage Hospital.—Miss Baker has obtained the appointment of Nurse-Matron at this Hospital. She has held the post of district nurse at Abinger, nurse at Dorchester, &c.

### For Reading to the Sick.

### REPENTANCE.

**Motto.**

Whatever may have been the past, however black and hideous,
It hath a present cure—repentance with amendment.

—*M. Tupper.*

**Verses.**

Because I knew not when my life was good,
And when there was a light upon my path,
But turned my soul perversely to the dark—
O Lord, I do repent!

Because I was impatient—would not wait,
But thrust mine impious hand across Thy threads,
And marred the pattern drawn out for my life—
O Lord, I do repent!

Because I called Good evil, Evil good,
And thought, I, ignorant, knew many things,
And deemed my weight of folly, weight of wit—
O Lord, I do repent!

Because Thou hast borne with me all this while,
Hast smitten me with love until I weep,
Hast called me as a mother calls her child—
O Lord, I do repent!

Because I spent the strength Thou gavest me
In struggle which Thou never didst ordain,
And have but dregs of life to offer Thee—
O Lord, I do repent!

Because I chose the thorns, and 'plained for flowers,
And pressed the sword points down upon my heart,
And moaned that they did hurt me, like a child—
O Lord, I do repent!

—*Sarah Williams.*

**Reading.**

When we consider that this is not the only life we have to live, and that we shall live in the next world according to our works in this, it becomes us to reflect seriously upon the past, especially when the silent hours of sickness afford us such abundant opportunity for self-examination. When we are healthy and strong, and occupied with the cares, the business, and the pleasures of this life, we far too often allow serious thoughts to be driven from our minds; but in sickness we are laid by in retirement, and have plenty of time given us by God for reflection. And when we thus examine ourselves we find that the Scripture is true which says: "There is none which doeth good and sinneth not." The lives of some persons are utterly careless and reckless, and we see their sin at once; but in many things we all offend God, even the best of us, and offend Him grievously. We shall soon be convinced of this truth if we only look into the past and see how many careless and evil thoughts, words and deeds, have been ours, how often "we have done those things which we ought not to have done, and left undone those things which we ought to have done." The holiest of Christians are always the most humble of men, because when they think of God and then think of themselves, they wonder how so good, so pure, so mighty a Being "dwelling in light unapproachable, and full of glory," can regard with favour such weak, wayward, wandering sinners as those who dwell on this earth. We never hear them say that they need no repentance, that they have no sin, that they have done no one any harm, and that they are as good as other people. No such language as this ever passes their lips, but, when they think of the mercies they receive from God, and their own spiritual condition, they cry, God be merciful to me, a sinner.

### The Book World for Women and Nurses.

[We invite Correspondence, Criticism, Enquiries, and Notes on Books likely to interest Women and Nurses. Address, Editor, The Hospital (Nurses' Book World), 428, Strand, W.C.]

A Threefold Mystery. A Tale of Monte Carlo. By Constance Serjeant. (London: Elliot Stock. 1894.)

The scene of this tale is laid in the Riviera, and very charmingly laid, too. Indeed, the *entourage* is the strong part of "A Threefold Mystery," an *entourage* so vividly and picturesquely described that the very fresh and enthusiastic manner in which it is written carries a certain contagion to its readers. Stories of the Riviera are not uncommon, especially romances which centre round Monte Carlo, but Miss Serjeant has succeeded in a task wherein many fail; she conveys to her readers through the medium of her pen something of the magic of southern sunshine and southern skies. There are indications throughout the book that the writer is perhaps one of no great experience, but those are deficiencies which are remediable with maturity. Miss Serjeant has the first quality of a writer, that is, the power of observation, a quality, alas! as a rule, more conspicuous by its absence than by its presence in the literature of to-day.

Names and their Meaning: A Book for the Curious. By Leopold Wagner. New and revised edition. (London: T. Fisher Unwin, Paternoster Square.)

Mr. Wagner's volume is a pleasant contribution, not merely to names, but, indirectly, to the social history of England. Going into the names, their origin and corruptions, of our streets and taverns, our towns and the various districts of them, Mr. Wagner brings before us the many changes—of politics and religion, of temper and habit—which have come about in the transformation of Celt and Saxon into the modern Englishman. It is a book full of curious information, suitable equally for reference and for passing pleasantly a leisure hour.

"Life's Little Ironies," and a Few Crusted Characters. By Thomas Hardy. (London: Osgood McIlvaine. 1894.)

Mr. Hardy has struck out a new line in "Life's Little Ironies," and in this volume of short stories we look in vain for that tender note of humanity which went so far to make the wide popularity of the author of "Far From the Madding Crowd" and "Tess of the D'Ubervilles." Tess was a creation; Leonora Frankland in "For Conscience Sake" is merely exhibited. It is as though Mr. Hardy would say to us, "I have shown you what I would wish men and women to be, now see them as they are," and of this latter class we have a beautifully-written example in "For Conscience Sake"—the most striking human study of the collection. Here we have the unsatisfactory truth brought home to us with deadly clearness that there is a time for everything, and even duty fulfilled too late brings its own unalterable Nemesis. The story is of a commonplace man who marries late in life a commonplace woman whom he had wronged twenty years before the story opens. The woman, Leonora, who by her own untiring efforts and successful personation of a widow had earned for herself and her daughter a coveted middle-class respectability, has the even tenour of her life disturbed by the reappearance of her lover after a silence of twenty years, offering her marriage. At first she refuses, and there is something repulsively hard, however natural, in the way she represents to him that she has no longer any personal desire to become his wife; secondly, that the township of Exonbury had unanimously recognised her claims to respectability; thirdly, that her daughter was about to become engaged to a highly-desirable young clergyman; and, fourthly, that he had come too late to be of any use to her. How he finally works upon her commonplace feelings into consenting to marry him, urged on by her reluctance, and how it all ended in failure is forcibly drawn. Romance is dead, but reality stands before us. Needless to say that all the sketches of "Life's Little Ironies" are admirable from a literary point of view, and whether they give pleasure or not is after all a matter of individual taste.

### MAGAZINES.

### The English Illustrated.

Poets of the gentler sex are to be congratulated on their champion in the "English Illustrated" of this month. Mr. Richard Le Gallienne, in "Woman Poets of the Day," has written the best article that has appeared on this now much canvassed subject. Whilst admitting that poetry has in the past been held almost exclusively as a male art, he points out the possibilities that women possess of entering the field, and shows that, in fact, the modern women have already entered it. Mr. Le Gallienne's article is written on the broad lines of poetry as a sexless art, and only treats the part that women have played in it where their work can stand for comparison side by side with the best types which have created for us a standard. He nevertheless criticises, and gives separate examples of the diction of most of the poetesses of the day (Mr. Le Gallienne objects to the title poetess). We are glad to note his appreciation of the talented authoress Mrs. Woods, whose terribly libelled portrait acts as frontispiece to the number of the magazine and the article. He expresses a wish to see another volume of Mrs. Woods poems, a desire we heartily echo. As in the case of Mrs. Woods, it is noticeable that many of the women poets are powerful writers of prose also. The women to whom our notice is specially drawn are Miss Jean Ingelow, Christina Rossetti, Dora Sigerson, Alice Meynell, and Katherine Tynan, whose portraits illustrate the article.

The first volume of The Yellow Book, an illustrated quarterly will be published on the 16th of this month. To judge by the list of authors and artists' names appearing among the contributors much may be expected of this new departure in the magazine world.

The April number of The Humanitarian contains, amongst others, two articles of especial interest. The one on Hypnotism, the other on The Physical Development of Women; the former of which, from the pen of Dr. George Kingsbury treats of hypnotism from a somewhat partial standpoint. The writer principally deals with one aspect of his subject, viz., the objections which have been, or are still, urged against the employment of hypnotic suggestion in therapeutics, his sole object being, he informs us, not so much the desire to convince his readers that hypnotism is a valuable remedial agent, as to remove objections which exist against according to it a fair, impartial, and critical trial. Geoffreys Mortimer's paper on The Physical Development of Women disclaims all feminine inability to sustained muscular exertion as being native. Such incapacity, according to our authority, is acquired, simply arising from want of practice. Mr. Mortimer's views on congenital physiological co-equality will, or should, be taken to heart by the fair sex, whose physique in 1894 contrasts but poorly with their savage sisters in their native states over the seas. Custom and not heredity, he insists, is the accountable agent in the strange inequality existing between woman in her civilised and uncivilised form.

